# Peripheral blood metabolome predicts mood change-related activity in mouse model of bipolar disorder

**DOI:** 10.1186/s13041-019-0527-3

**Published:** 2019-12-10

**Authors:** Hideo Hagihara, Tomoyasu Horikawa, Yasuhiro Irino, Hironori K. Nakamura, Juzoh Umemori, Hirotaka Shoji, Masaru Yoshida, Yukiyasu Kamitani, Tsuyoshi Miyakawa

**Affiliations:** 1 Division of Systems Medical Science, Institute for Comprehensive Medical Science, Fujita Health University, Kutsukake-cho, Toyoake, Aichi 470-1192 Japan; 2 Department of Neuroinformatics, ATR Computational Neuroscience Laboratories, Kyoto, 619-0288 Japan; 3 Division of Evidence-based Laboratory Medicine, Kobe University, Graduate School of Medicine, Kobe, 650-0017 Japan; 4 Division of Metabolomics Research, Department of Internal Related, Kobe University Graduate School of Medicine, Kobe, 650-0017 Japan; 5 Division of Gastroenterology, Department of Internal Medicine, Kobe University Graduate School of Medicine, Kobe, 650-0017 Japan; 6 Graduate School of Informatics, Kyoto University, Kyoto, 606-8501 Japan

## Abstract

Bipolar disorder is a major mental illness characterized by severe swings in mood and activity levels which occur with variable amplitude and frequency. Attempts have been made to identify mood states and biological features associated with mood changes to compensate for current clinical diagnosis, which is mainly based on patients’ subjective reports. Here, we used infradian (a cycle > 24 h) cyclic locomotor activity in a mouse model useful for the study of bipolar disorder as a proxy for mood changes. We show that metabolome patterns in peripheral blood could retrospectively predict the locomotor activity levels. We longitudinally monitored locomotor activity in the home cage, and subsequently collected peripheral blood and performed metabolomic analyses. We then constructed cross-validated linear regression models based on blood metabolome patterns to predict locomotor activity levels of individual mice. Our analysis revealed a significant correlation between actual and predicted activity levels, indicative of successful predictions. Pathway analysis of metabolites used for successful predictions showed enrichment in mitochondria metabolism-related terms, such as “Warburg effect” and “citric acid cycle.” In addition, we found that peripheral blood metabolome patterns predicted expression levels of genes implicated in bipolar disorder in the hippocampus, a brain region responsible for mood regulation, suggesting that the brain–periphery axis is related to mood-change-associated behaviors. Our results may serve as a basis for predicting individual mood states through blood metabolomics in bipolar disorder and other mood disorders and may provide potential insight into systemic metabolic activity in relation to mood changes.

## Introduction

Mood naturally changes over time with variable amplitude and frequency, sometimes in an infradian (a cycle > 24 h) manner [1, 2]. This is often accompanied by changes in physical activity [3]. In bipolar disorder, unusual swings in mood and activity levels from depressive to manic states are core phenomenological and clinical features. Such exaggerated fluctuations in mood and the related activity can cause significant distress and/or social and occupational impairment, often leading to high direct and indirect health care costs [4, 5]. Currently, the clinical assessment and management of these conditions mainly rely on clinicians’ interview and patients’ subjective description. To overcome the potential subjective biases, developing objective and quantitative measures is expected to compensate for the current procedures. To this end, substantial efforts have been invested into predicting mood states of patients with bipolar disorder based on biological features, such as heartbeat [6], spontaneous speech [7], and neural activity measured with functional magnetic resonance imaging [8]. Meanwhile, molecular omics approaches, such as metabolomics and proteomics, with which hundreds of molecules can be targeted simultaneously, are being applied to gain more complete profiles of a wide range of biological conditions and diseases [9, 10]. However, such molecular omics-based approaches have not been well examined to predict mood states. While mood disorders are considered to be brain diseases, the use of peripheral samples has been desirable for clinical purposes, as the opportunity to obtain human brain tissues is limited [11].

Previous studies have attempted to detect biomarkers for mood disorders in the blood by comparing the metabolomes of patients to control subjects [11–13]. It is still not well understood whether or to what extent metabolomic alterations in peripheral blood are related to the shift of mood states. This may, in part, be due to the lack of animal models that exhibit behavioral phenotypes associated with infradian changes in mood states. We previously found that mice with the heterozygous knockout of the alpha-isoform of calcium/calmodulin-dependent protein kinase II (*Camk2a*^+/−^ mice) exhibit various dysregulated behaviors, including exaggerated cyclic mood-change-associated activity in an infradian manner, in which locomotor activity (LA) spontaneously and recurrently increases and decreases in a period of approximately 10–20 days [14, 15]. Importantly, changes in the LA were correlated with changes in depression-like and anxiety-like behaviors [14], suggesting that *Camk2a*^+/−^ mice are useful as an animal model which shows similar infradian oscillations of mood to those found in patients with bipolar disorder. CAMK2A is one gene suggested to be a candidate for bipolar disorder [16–18], and decreased CAMK2A mRNA has been found in the prefrontal cortex of patients with the disorder [19]. *Camk2a*^+/−^ mice also harbor brain endophenotypes relevant to bipolar disorder, such as neuronal hyperexcitability [20] and immaturity [15, 21–23] in the hippocampus and decreased brain pH [24]. These findings suggest that *Camk2a*^+/−^ mice provide a model for bipolar disorder which has good face and construct validity [25].

Using the *Camk2a*^+/−^ mice, we previously showed that gene expression patterns in the hippocampus, a brain region implicated in mood regulation, could retrospectively predict the LA level of individual mice by using statistical learning algorithm [14]. We used cross-validated multivariate regression models to ensure the generalization ability of our prediction models. Generalization ability is the capacity to predict unseen samples, and ensuring and improving the generalization ability of models are believed to be crucial for practical use, including identification of subpopulations among patients with mood disorders [26]. In the present study, using this strategy, we sought to determine whether metabolome patterns in peripheral blood can predict individual LA levels of the mice, which would have potential for future translational applications. We also investigated whether gene expression levels in the brain can be predicted by peripheral blood metabolome patterns, aiming to gain insights into the link between systemic metabolic pathways and the regulation of brain gene expression in relation to the infradian mood changes.

## Materials and methods

### Animals

Thirty-seven adult (> 8 weeks old) male *Camk2a*^+/−^ mice were used [14, 15, 27]. Every effort was made to minimize the number of animals used.

### Locomotor activity monitoring and blood sampling

**Fig. 1:**
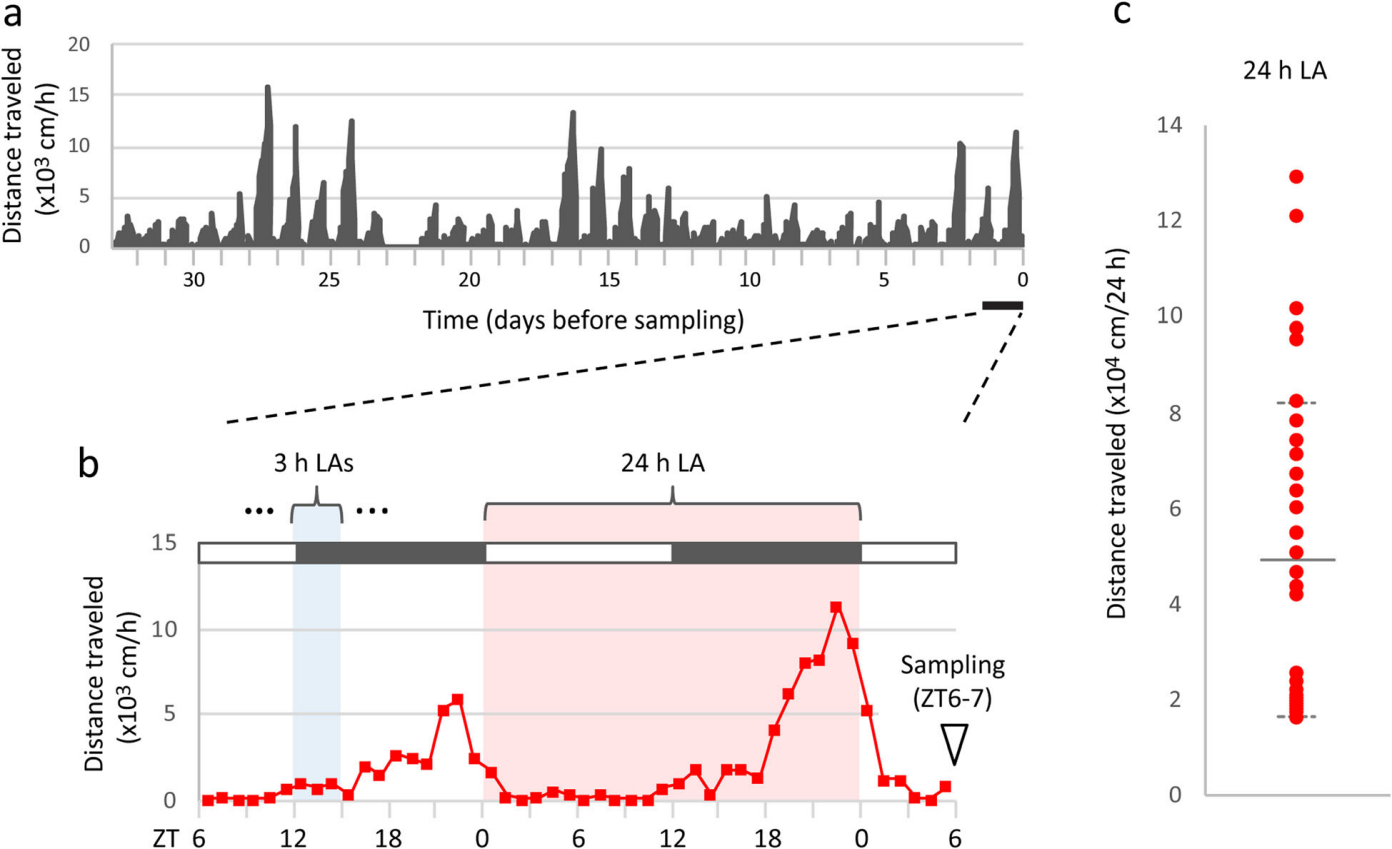
Experimental workflow. **a** LA was longitudinally monitored, and blood was taken from each mouse ZT6–7. **b** 24 h LA, distance traveled during the 24 h before ZT0 on the sampling day; 3 h LAs, distances traveled of every 3 h window before sampling (ZT6). Black and white bars indicate dark and light period, respectively. **c** Variations in 24 h LA. Each dot indicates an individual mouse. Solid lines indicate average 24 h LA and dashed lines indicate the standard deviations for 35 mice. LA, locomotor activity; ZT, zeitgeber time

The LA data of *Camk2a*^+/−^ mice analyzed in this study were the same as was used in a previous study [14]. Mice were singly housed with a 12 h light/dark cycle (lights on at 7:00 a.m.) and access to food and water ad libitum. The monitoring of LA and blood sampling was performed as previously described [14]. Briefly, LA was monitored for 72–82 days through a system that automatically analyzes the distance traveled by a mouse in its home cage [15]. Peripheral blood and brain tissue [14], was collected at zeitgeber time (ZT) 6–7 (ZT0, lights on; ZT12, lights off) from mice with short or long distances traveled (assessed by distance traveled during the 24 h before ZT0 on the sampling day; Fig. 1a, b). In this way, mice were selected for the sampling such that their 24 h LA levels varied among the 37 mice (Fig. 1c). The 24 h LA was defined as distance traveled during the 24 h between ZT0 on the day before the sampling and ZT0 on the day of sampling. The 3 h LA was defined as distance traveled in every 3 h window before sampling (ZT6) (Fig. 1b).

Mice were removed from the home cage, and immediately euthanatized by cervical dislocation. Blood was collected from the neck in tubes with Na-heparin and centrifuged (2200×*g* at 4 °C for 10 min) to prepare plasma. Plasma samples were stored at − 80 °C until use.

### Gas chromatography mass spectrometry (GC/MS) analysis of blood samples

GC/MS analysis was performed as described previously [28] using a GCMS-QP2010 Ultra (Shimazu, Kyoto, Japan) with a fused silica capillary column (CP-SIL 8 CB low bleed/MS; 30 m × 0.25 mm inner diameter, film thickness 0.25 μm; Agilent Technologies, Palo Alto, CA, USA). Data processing was performed as described previously [28]. 2-isopropylmalic acid was used as an internal standard. Two mice whose metabolite contents could not be measured due to unknown reasons were excluded from further analyses. Hence, LA and metabolome data from 35 mutant mice were processed for correlation and prediction analyses (Additional file 2: Table S1), as described below.

### Brain gene expression data

Expression microarray data of the hippocampal dentate gyrus [29] was obtained from the 35 mice mentioned above. We used data that has been deposited at the Gene Expression Omnibus (GEO; accession number GSE68293) [14]. The log_2_-transformed expression values were used for the prediction analyses.

### Construction of models to predict LA

The linear regression algorithm was used to predict LA (24 h LA and 3 h LAs; Fig. 1) and gene expression levels of individual mice from peripheral blood metabolome patterns using MATLAB [14]. The prediction accuracy was evaluated by the leave-one-out cross-validation method and the feature selection for the prediction was conducted by the nested cross-validation method. Calculation details are included in the Additional file 1: Supplementary Materials and Methods.

### Pathway enrichment analysis

To determine the characteristics of metabolites of interest, we used the default settings in MetaboAnalyst (version 4.0; http://www.metaboanalyst.ca/) [30], a comprehensive, web-based tool for metabolomics analysis and interpretation. Some feature names that had not been recognized in the query were modified if necessary, as suggested in the instructions.

## Results

### Locomotor activity can be predicted from blood metabolomic profiles

**Fig. 2:**
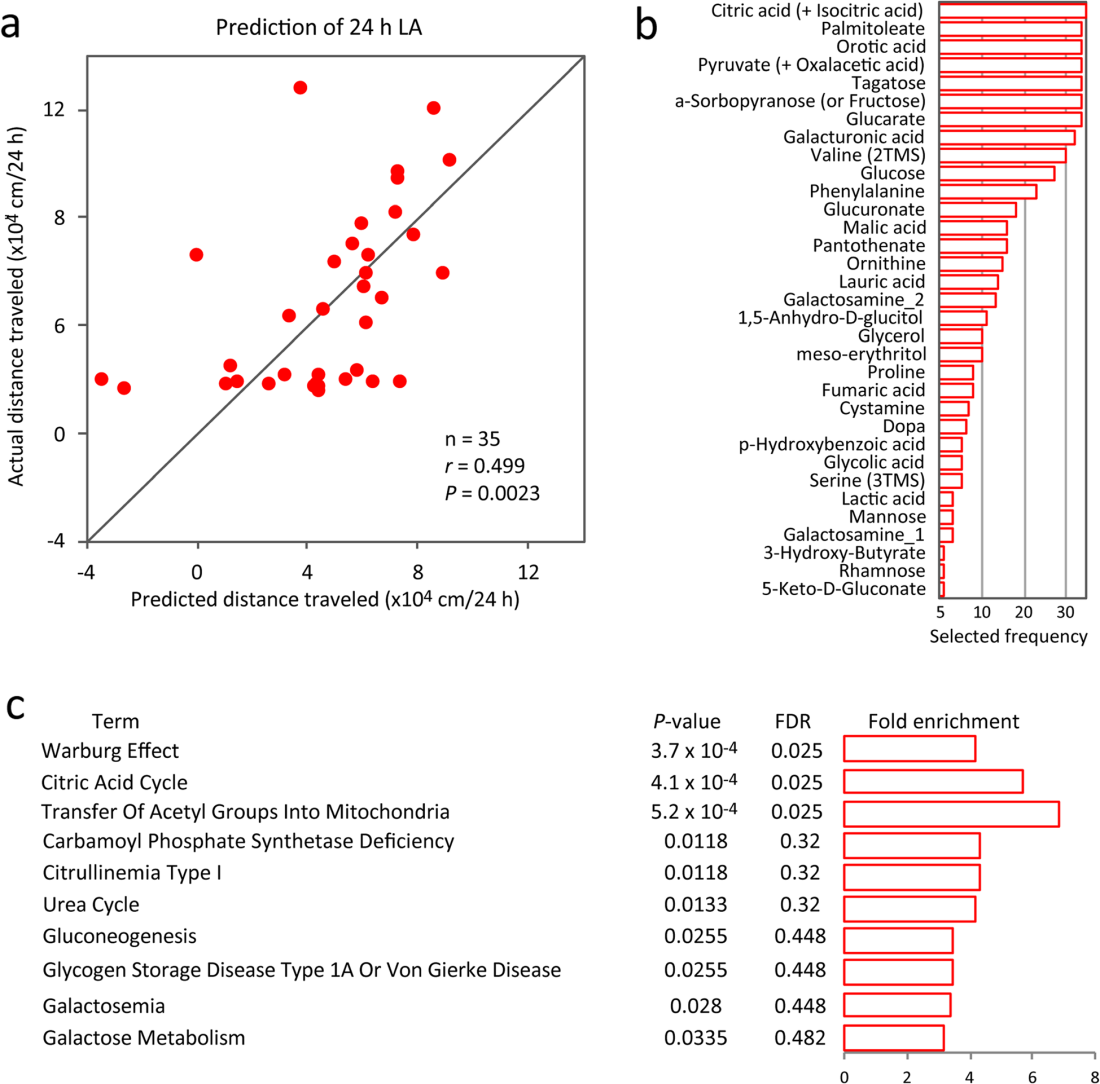
Metabolome patterns in the peripheral blood can predict 24 h LA in *Camk2a*^+/−^ mice. **a** Scatter plot showing a significant correlation between predicted and actual 24 h LA (*N* = 35 mice). **b** Feature preference for constructing the 24 h LA prediction model. **c** Results of pathway enrichment analysis for the metabolites used for constructing 24 h LA prediction model. The statistically enriched terms with raw *P*-value below 0.05 are shown. LA, locomotor activity

Of the 106 metabolites tested, the concentrations of 16 features were correlated with 24 h LA (*P* < 0.05, Pearson’s correlation; Additional file 1: Figure S1). None of these survived false discovery rate correction for multiple tests (FDR; *q* value < 0.1). To determine whether the metabolome patterns in peripheral blood could retrospectively predict the 24 h LA of individual mice, we performed linear regression analysis with the nested cross-validation method [14]. Independent sets of mice were used for feature selection from entire set of 106 metabolites and model fitting in the inner cross-validation loop and testing the model performance in the outer cross-validation loop. Statistical evaluation of the prediction accuracy of the model revealed a significant correlation between the actual and the predicted 24 h LA (Fig. 2a), indicating that the metabolome patterns in the peripheral blood quantitatively predict the 24 h LA of individual mice. Thirty-three out of the 106 metabolites were selected to build the successful prediction model (Fig. 2b, Additional file 1: Figure S1).

### Mitochondrial alterations implicated in infradian oscillatory LA

The prediction algorithm we used identified metabolome signatures related to infradian oscillatory LA by weighting metabolites according to their individual predictive strength (Fig. 2b). Next, we examined the weighted metabolites that were selected to predict the 24 h LA to gain insight into metabolic alterations related to changes in infradian oscillatory LA. Pathway enrichment analysis using a bioinformatics tool MetboAnalyst [30] revealed that the set of metabolites exhibited enrichments in the Warburg effect, the citric acid cycle, and mitochondria-related pathway. These survived multiple comparison correction (FDR *q* value < 0.05; Fig. 2c). Interestingly, all the terms were to mitochondrial functions (see Discussion), suggesting that mitochondrial function may change concomitantly with infradian oscillatory LA.

### Peripheral blood metabolome predicts LA levels for longer time periods

**Fig. 3:**
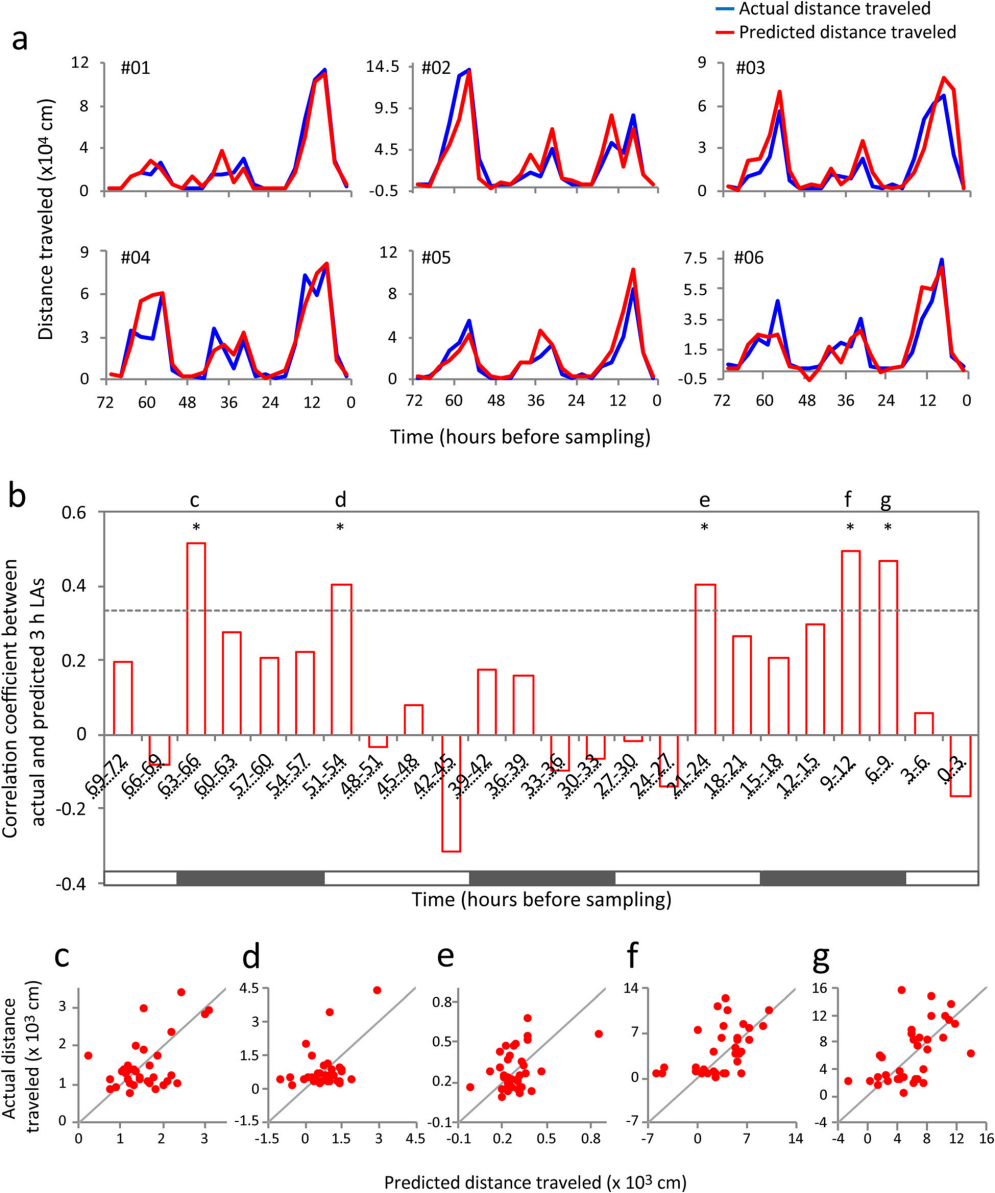
Prediction of 3 h LAs during the 3 days before sampling. **a** The prediction results of 6 mice are shown; the results of the remaining 29 mice are shown in Additional file 1: Figure S2. **b** Correlation coefficients between the actual and the predicted 3 h LAs in each time window. Dashed lines indicate *P* value of 0.05 and asterisks indicate significant correlations between actual and predicted 3 h LAs after FDR correction (*q* value < 0.1). Black and white bars indicate dark and light periods, respectively. **c**–**g** Scatter plots of predicted and actual 3 h LAs of each mouse at the time windows indicated in b. LA, locomotor activity

To investigate whether metabolome patterns in peripheral blood could predict the LA of the past several days, we constructed models for predicting the LA within the 3 days before sampling using a sliding time window (window size: 3 h, step size: 3 h; yielding 24 time windows) (3 h LAs; Fig. 1b). The actual and the predicted 3 h LAs were similar within the past 3 days in most mice, while differences between them were apparent in some mice (Fig. 3a, Additional file 1: Figure S2). Overall, statistical evaluation of prediction accuracy of the models detected significant correlations between the actual and the predicted 3 h LAs in 5 of the 24 time windows after FDR correction (time windows of 6–9 h: *r* = 0.46, *P* = 0.0049; 9–12 h: *r* = 0.49, *P* = 0.0025; 21–24 h: *r* = 0.41, *P* = 0.016; 51–54 h: *r* = 0.41, *P* = 0.016; 63–66 h: *r* = 0.51, *P* = 0.0017; Fig. 3b–g). The oldest time window with a significant correlation was from 63 to 66 h before sampling. These results suggest that multivariate patterns of the metabolome in the peripheral blood hold information about LA of at least the past 3 days. Out of the five time windows mentioned above, metabolites selected were similar in 3 h LA predictions of 6–9 h and 9–12 h time windows (as well as the 24 h LA prediction), and differed in other 3 h time windows (Additional file 1: Figure S3). Metabolites selected for the prediction of the oldest 3 h time window (63–66 h LA) were associated with aspartate-related pathways (unadjusted *P* < 0.05; Additional file 1: Figure S4), which differed from those for 24 h LA prediction (Fig. 2c).

### Hippocampal expression levels of the *Arntl* gene can be predicted from peripheral blood metabolome

**Fig. 4:**
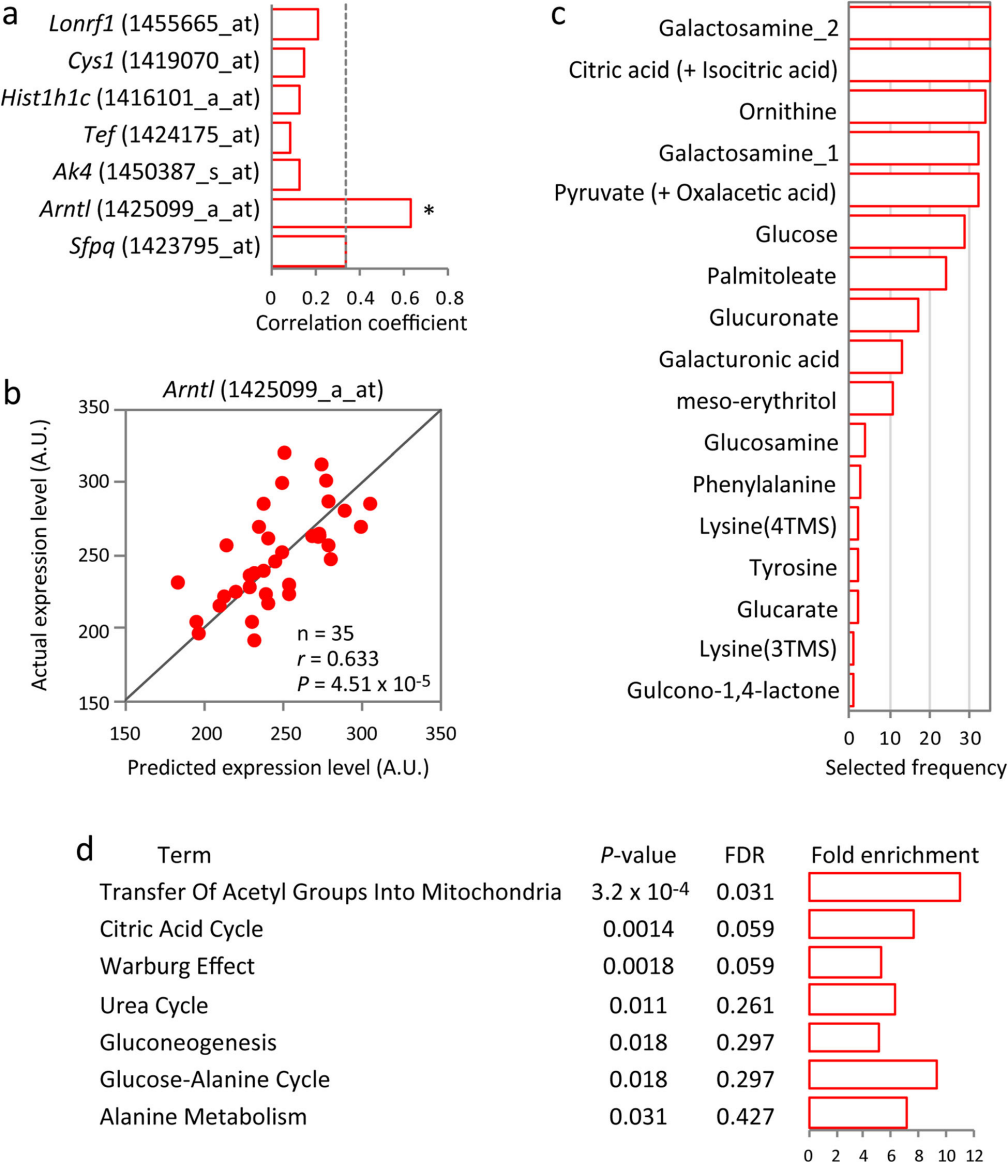
Prediction of hippocampal gene expression from peripheral blood metabolome. **a** Genes processed for this prediction analysis were provided from a previous study by Hagihara et al. [14]. The correlation coefficients between actual and predicted expression levels of each gene are shown. Dashed lines indicatea a *P*-value of 0.05 and asterisks indicates significant correlations after Bonferroni correction for multiple testing (significance level: *P* < 0.00714 = 0.05/7). **b** Scatter plot showing significant correlations between actual and predicted expression level of *Arntl*. The correlation was not due to outliers. **c** Feature preference for constructing the prediction model of *Arntl* expression level. **d** Results of pathway enrichment analysis for the metabolites used for constructing the prediction model of *Arntl* expression level. The statistical enriched terms with raw *P*-value below 0.05 are shown

The regulation of mood is thought to involve the hippocampus [31, 32] and hippocampal expression of some circadian genes are closely related to infradian oscillation of LA in *Camk2a*^+/−^ mice [14]. Consequently, using a prediction approach, we asked whether metabolome patterns in peripheral blood are related to hippocampal expression of circadian genes in relation to mood-change-associated activity. We used microarray-based gene expression data in the hippocampus of *Camk2a*^+/−^ mice that we had previously obtained from the same mice whose blood metabolome data was analyzed above [14]. Cross-validated prediction models based on the blood metabolome data were constructed to predict expression levels of genes, focusing on seven circadian-related genes (*Lonrf1*, *Cys1*, *Hist1h1c*, *Tef*, *Ak4*, *Arntl*, and *Sfpq*), which were among the genes that were selected to predict the 24 h LA of *Camk2a*^+/−^ mice [14]. Of the seven genes tested, *Arntl* and *Sfpq* expression levels could be predicted by blood metabolome patterns, which was determined by correlations between actual and predicted expression levels (*P* < 0.05), and *Arntl* survived correction for multiple testing (Fig. 4a, b). Metabolites used for the successful prediction of hippocampal *Arntl* expression levels showed enrichment for mitochondria-related pathways (Fig. 4c, d).

## Discussion

In this study, we demonstrated for the first time that peripheral blood metabolomics in combination with a statistical learning algorithm can predict individual levels of mood-change-related activity using an animal model showing exaggerated infradian rhythm. It could be suggested that this result is simply a consequence of the metabolite secretions affected by LA immediately before sampling. However, considering that blood metabolome patterns could not predict LA during the 0–6 h immediately before sampling (Fig. 3b), this cannot be considered a major factor in determining the blood metabolome patterns. Thus, blood metabolome patterns may hold information about infradian states of LA oscillation during specific time windows that are several hours or days apart from the sampling time.

Our prediction approach highlighted potential alterations in mitochondria metabolism concomitant with infradian mood change-related activity. The Warburg effect is a shift in metabolism towards aerobic glycolysis away from oxidative phosphorylation even in the presence of oxygen which is induced through mitochondrial dysfunction. It was first described in cancer, and studies of both patients’ brains and of mouse models have recently been suggested that it could be involved in several psychiatric disorders [24, 33]. Alterations in metabolites related to the citric acid cycle have been found in peripheral blood and cerebrospinal fluid of patients with bipolar disorder [11, 34]. While these studies detected trait-related alterations and support the mitochondria hypothesis of bipolar disorder [35–38], our present study suggests that mitochondrial alterations are related to state-related alterations, or changes in mood states. However, the causal relationship between the mitochondria-related metabolic alterations in the blood and changes in LA levels remains unclear. Regarding state-related alterations, a recent meta-analysis indicated that blood levels of some inflammatory cytokines, such as IL-6 and sIL-6R, were increased in manic and euthymic states but not the depressive state compared to controls [39]. Other study has shown that blood levels of IL-6 and IL-2 are positively correlated with mood symptoms, as indicated by the Young Mania Rating Scale [40]. These findings suggest that inflammatory conditions could alter depending on affective states in bipolar disorder, with an enhanced inflammatory-related signature in manic states. Supporting these observations, we found that pyruvate, a metabolite suggested to be an endogenous anti-inflammatory molecule [41], was negatively correlated with LA in *Camk2a*^+/−^ mice, and relatively low in manic-like states (Additional file 1: Figure S1). Although increased blood levels of pyruvate has implicated mitochondrial dysfunction in bipolar disorder, its significance on the pathogenesis of the disorder has remained unclear [11]. Considering the findings of the present study and those of studies discussed above, mitochondrial destabilization may be intricately associated with pro- and anti-inflammatory balance in relation to changes in mood states as well as to the pathogenesis of the disorder [42].

We found that blood metabolome patterns predicted 3 h LA at 3 days before sampling (63–66 h LA; Fig. 3b, c), and metabolites selected for the prediction showed enrichments in aspartate-related pathways (Additional file 1: Figure S4). Alterations in aspartate metabolism have been suggested in the brain and blood of patients with bipolar disorder [11, 43]; however, their relations with mood changes are unknown. Considering that metabolites selected for the prediction of 3 h LA at 63–66 h before sampling and the related metabolic pathways were different from those for 24 h LA prediction (Fig. 2, Additional file 2: Table S1), levels of a distinct set of blood metabolites and the metabolic pathways might retain information about LA during different and specific time windows.

We also found that the peripheral blood metabolome can predict expression levels of the *Arntl* gene in the hippocampus, a brain region associated with mood regulation [31, 32], and that the metabolites that successfully predicted these levels showed enrichment for mitochondria-related pathways. These results suggest that systemic alterations in mitochondrial metabolism and hippocampal expression of *Arntl*, a candidate gene for bipolar disorder [44–46], may serve as a link for brain–periphery interactions in relation to mood-change-associated locomotor activity. Blood metabolites that change according to infradian cyclic locomotor activity could be associated not merely with the physical activity levels, but also potentially with hippocampal function in mood regulation via concomitant expression changes of the bipolar disorder candidate gene.

In conclusion, the present study provided evidence that metabolome patterns in peripheral blood could be used as predictors of states of mood-change-related activity in this mouse model. As a limitation of this study, a recent history of physical activity might change peripheral metabolome patterns independent of the mood state, as intense or moderate exercise might affect mitochondria-related pathways in the brain, heart, and other organs in mice [47, 48]. Among *Camk2a*^+/−^ mice, we previously showed that levels of infradian oscillatory LA in the home cages were associated with anxiety-like and depression-like behaviors [14]. Although it is most likely that the LA state in the home cage reflects a certain state of mood in *Camk2a*^+/−^ mice, the effects of physical activity independent of the mood state on blood metabolome patterns should be considered as potential confounding factors in this study. This study, although limited to an animal model, may have potential for translational applications to human studies, as peripheral blood can be collected from living subjects in a minimally invasive way. States of mood and the related behaviors in human, especially patients with mood disorders, could potentially be retrospectively and prospectively predicted with blood samples through methods analogous to those used here. This may help to develop novel biologically-based methods for the diagnosis and treatment of bipolar disorder. To this end, further studies are needed to determine the most appropriate samples to collect (e.g., blood, saliva, urine, or skin), the appropriate processing methods (e.g., metabolomics, proteomics, or transcriptomics) and to optimize the prediction algorithm [49].

## Supplementary information

**Additional file 1. **Supplementary Materials and Methods. **Figure S1.** Correlations between 24 h LA and levels of metabolites that were used for the prediction of 24 h LA. **Figure S2.** Prediction results of 3 h LAs from metabolome patterns in peripheral blood of *Camk2a*^+/−^ mice. Prediction of 3 h LAs during the 3 days before sampling for 29 mice. LA, locomotor activity. **Figure S3.** Metabolites used in prediction models that significantly predicted 3 h LA. Hierarchical clustering of metabolites based on their frequencies selected to build cross-validated prediction models for each time window of LA using R software (http://www.r-project.org/). LA, locomotor activity. **Figure S4.** Pathway enrichment analysis of the metabolites used for constructing a prediction model of 3 h LA at 63–66 h before sampling. The statistically enriched terms with raw *P*-values below 0.05 are shown.

**Additional file 2: Table S1.** The raw data used for this study.

## Data Availability

We provided the raw data used for this study in Additional file 2: Table S1.

## Abbreviations

Ak4: Adenylate kinase 4
Arntl: Aryl hydrocarbon receptor nuclear translocator-like
Camk2a: alpha-isoform of calcium/calmodulin-dependent protein kinase II
Cys1: Cystin 1
FDR: False discovery rate
GC/MS: Gas chromatography mass spectrometry
Hist1h1c: Histone cluster 1, H1c
IL-6: Interleukin-6
LA: Locomotor activity
Lonrf1: LON peptidase N-terminal domain and ring finger 1
Sfpq: Splicing factor proline/glutamine rich
sIL-6R: soluble interleukin-6 receptor
Tef: Thyrotroph embryonic factor
ZT: Zeitgeber time
