# Supplementary material for: Peripheral blood metabolome predicts mood change-related activity in mouse model of bipolar disorder
Source: Mol Brain. 2019 Dec 10;12:107. doi: 10.1186/s13041-019-0527-3 (PMC6902552; doi:10.1186/s13041-019-0527-3)
Supplement: Supplementary file 1 — Additional file 1. Supplementary Materials and Methods. Figure S1. Correlations between 24 h LA and levels of metabolites that were used for the prediction of 24 h LA. Figure S2. Prediction results of 3 h LAs from metabolome patterns in peripheral blood of Camk2a+/− mice. Prediction of 3 h LAs during the 3 days before sampling for 29 mice. LA, locomotor activity. Figure S3. Metabolites used in prediction models that significantly predicted 3 h LA. Hierarchical clustering of metabolites based on their frequencies selected to build cross-validated prediction models for each time window of LA using R software (http://www.r-project.org/). LA, locomotor activity. Figure S4. Pathway enrichment analysis of the metabolites used for constructing a prediction model of 3 h LA at 63–66 h before sampling. The statistically enriched terms with raw P-values below 0.05 are shown. [file 13041_2019_527_MOESM1_ESM.pdf]

Title: Peripheral blood metabolome predicts mood change-related activity in mouse model of bipolar disorder

Running title: Blood metabolome predicts mood-related behavior

Authors: Hideo Hagihara, PhD[1]; Tomoyasu Horikawa, PhD[2]; Yasuhiro Irino, PhD[3]; Hironori K. Nakamura, PhD[1]; Juzoh Umemori, PhD[1]; Hirotaka Shoji, PhD[1]; Masaru Yoshida, MD, PhD[4,5]; Yukiyasu Kamitani, PhD[2,6]; Tsuyoshi Miyakawa, PhD[1*]

Affiliations:

[1]Division of Systems Medical Science, Institute for Comprehensive Medical Science, Fujita Health University, Toyoake 470-1192, Japan.

[2]ATR Computational Neuroscience Laboratories, Kyoto 619-0288, Japan.

[3]Division of Evidence-based Laboratory Medicine, Kobe University, Graduate School of Medicine, Kobe 650-0017, Japan.

[4]Division of Metabolomics Research, Department of Internal Related, Kobe University Graduate School of Medicine, Kobe 650-0017, Japan.

[5]Division of Gastroenterology, Department of Internal Medicine, Kobe University Graduate School of Medicine, Kobe 650-0017, Japan.

[6]Graduate School of Informatics, Kyoto University, Kyoto 606-8501, Japan.

*Corresponding Author: Tsuyoshi Miyakawa, Division of Systems Medical Science, Institute for Comprehensive Medical Science, Fujita Health University, Kutsukake-cho, Toyoake, Aichi 470-1192, Japan, Tel: +81-562-93-9376, Fax: +81-562-92-5382, E-mail: miyakawa@fujita-hu.ac.jp

Supplementary Information

Supplementary Materials and Methods

Construction of models to predict LA. The linear regression analysis with the nested cross-validation method was performed to predict LA (24-LA and 3-hr LAs; Fig. 1) from metabolite expression values for individual mice using MATLAB. The nested cross-validation consists of an inner loop for model fitting and parameter optimization and an outer loop for assessment of prediction performance. From the analyzed metabolite expression dataset of 35 mice, one sample was selected and excluded to serve as test data for the outer cross-validation. Then, one of the remaining samples was randomly chosen and put aside to serve as test data for the inner cross-validation. In the inner loop, a linear regression model was trained on the remaining 33 samples to find the optimal number of features. For this, correlations between LA data and metabolite expression data were calculated, and the number of features was increased stepwise from 1 to 32 according to the absolute value of the calculated correlation coefficient. For each feature number, the linear regression model was trained 33 times (the number of samples in the inner loop), in which every sample except for the outer test samples was used once as the inner test sample. The accuracy of the inner test samples was assessed, and the optimal feature number was used to train classifiers in the outer loop. In the outer cross-validation, the linear regression model was trained on all samples except for the outer test samples using the optimal number of features from the inner loop, and the outer test samples were predicted. This was repeated 35 times, in which all samples were chosen once as the outer test data. Prediction performance was analyzed by evaluating correlation coefficients between the predicted and actual values for the 35 mice. Essentially the same methods as shown above were used to predict gene expression levels from blood metabolome data. Thus, individual metabolites could be selected ≤35 times in each cross-validated prediction model. Metabolites selected at least once in the model were considered as predictive metabolites.

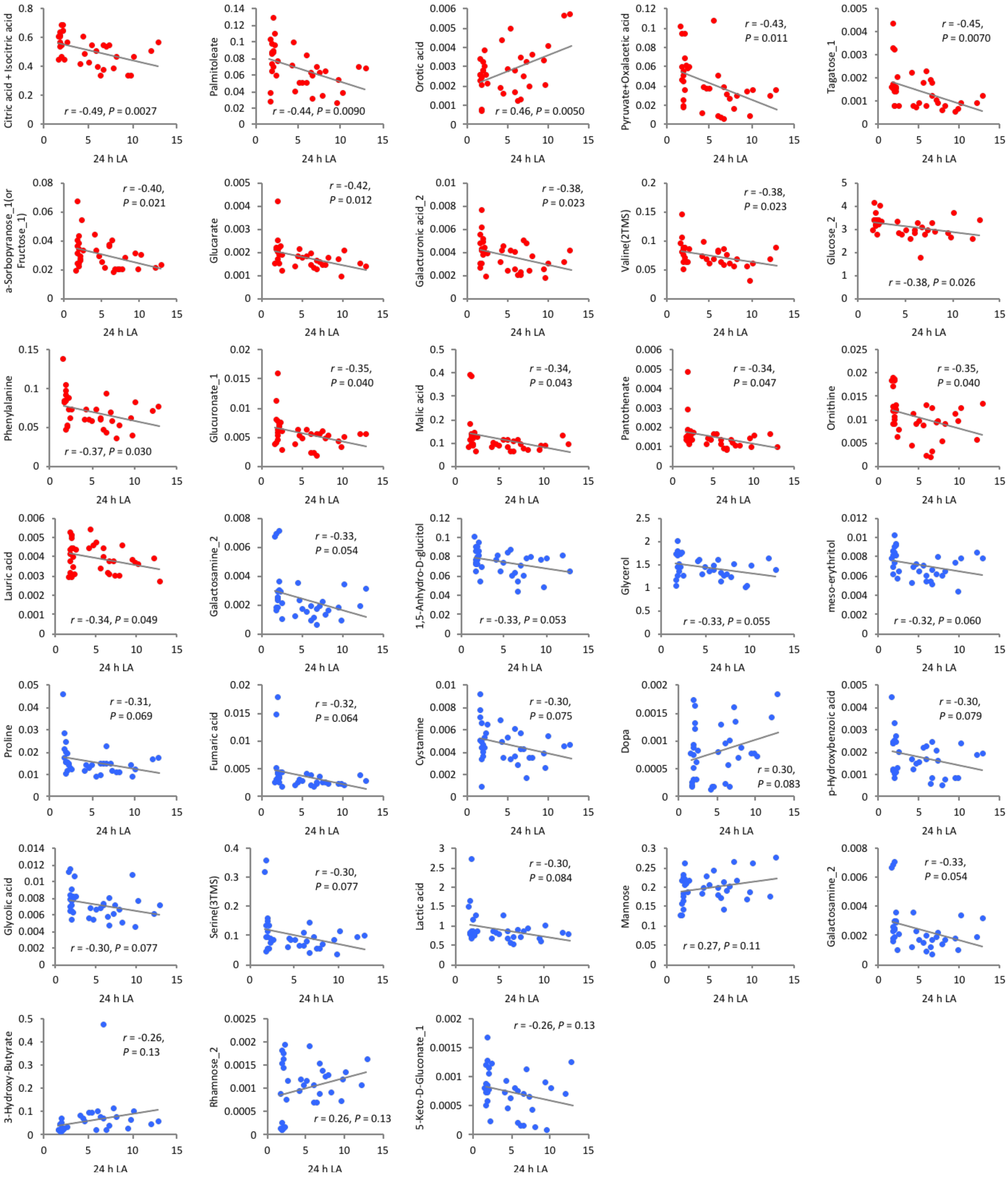

Figure S1. Correlations between 24 h LA and levels of metabolites that were used for the prediction of 24 h LA. Scatter plots showing correlations between 24 h LA and each metabolite concentration in the blood. Significant correlations with raw *P*-value < 0.05 are shown in red plots and non-significant correlations in blue plots. LA, locomotor activity.

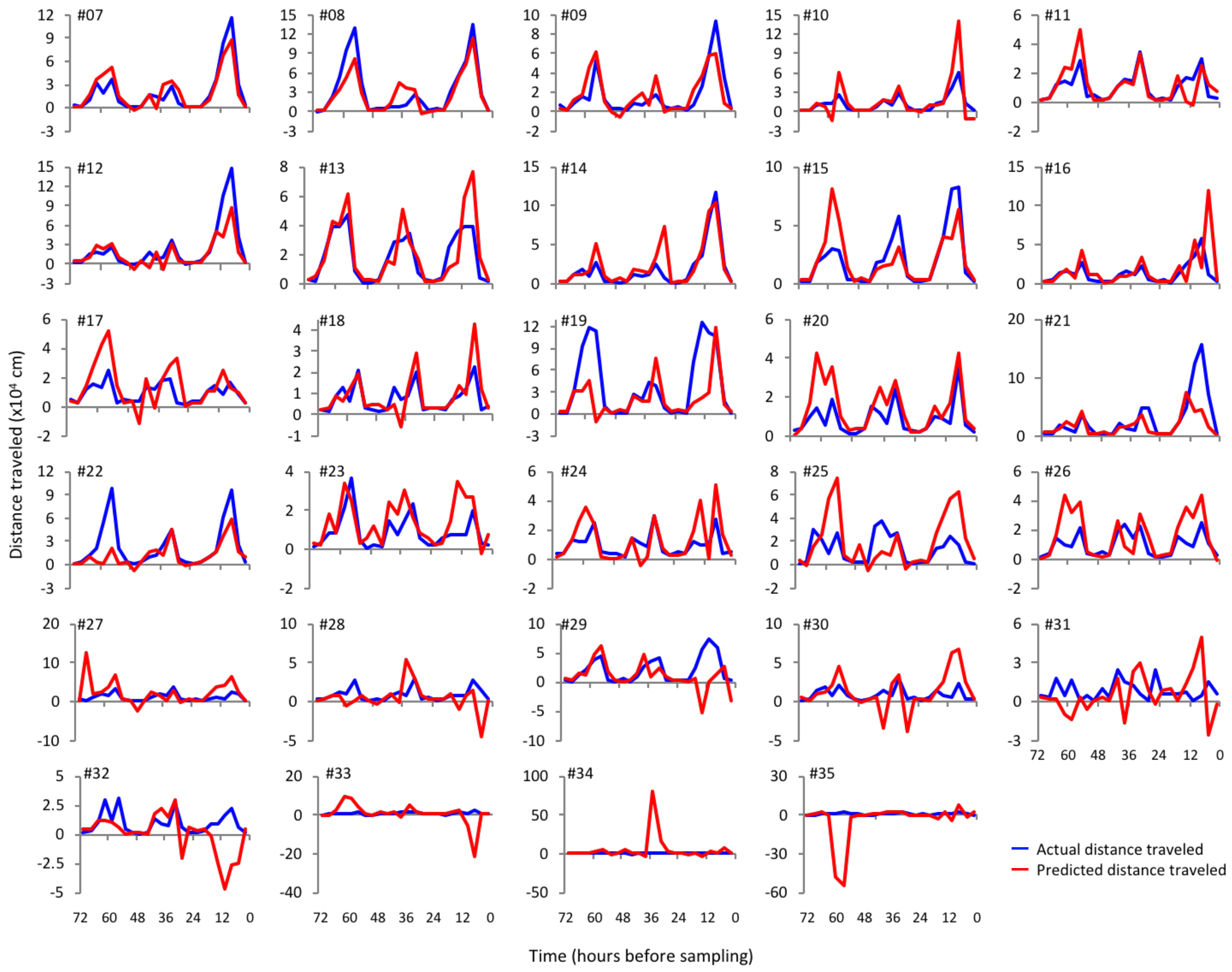

Figure S2. Prediction results of 3 h LAs from metabolome patterns in peripheral blood of *Camk2a*+/- mice. Prediction of 3 h LAs during the 3 days before sampling for 29 mice. LA, locomotor activity.

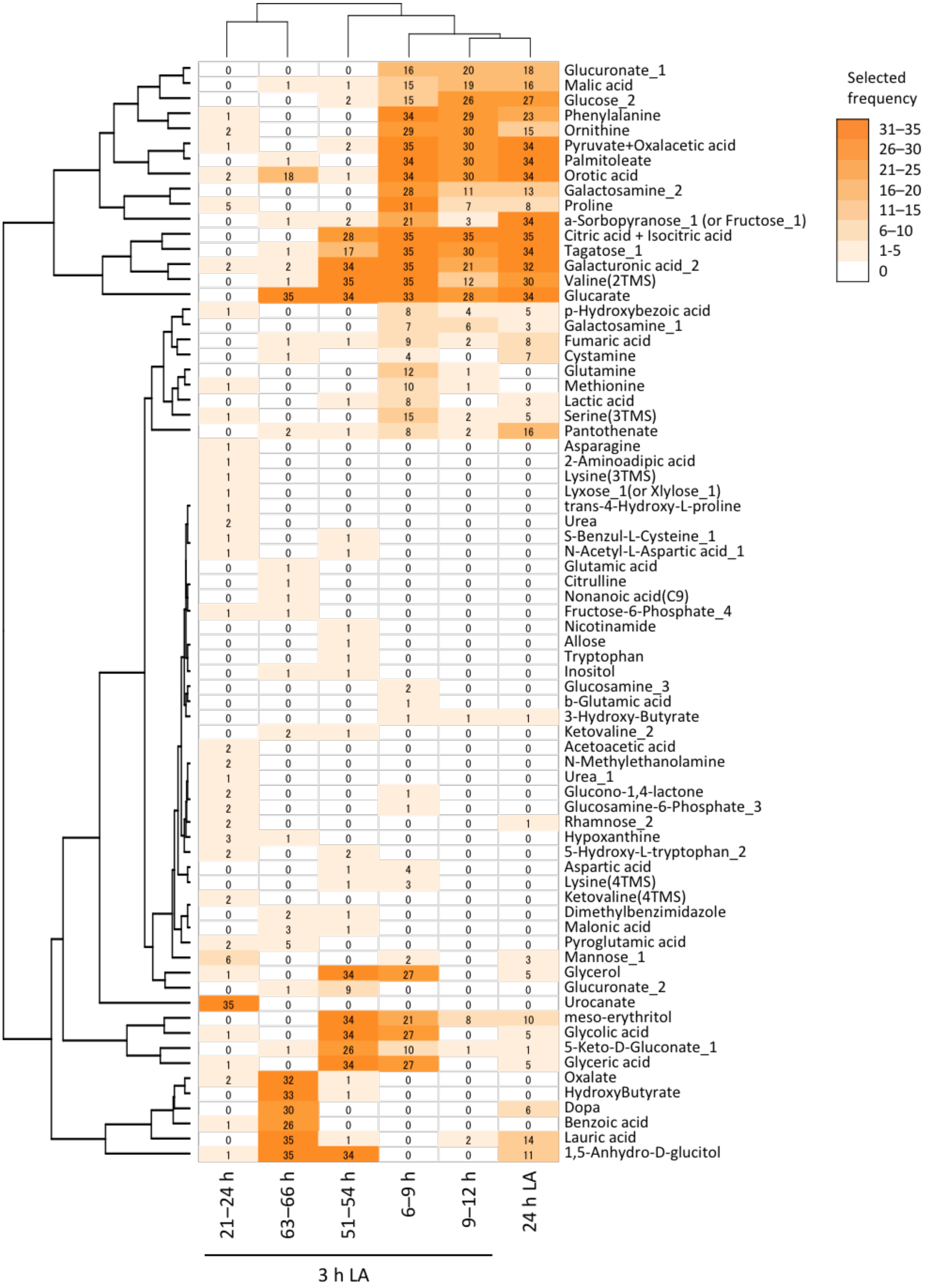

Selected frequency
31–35
26–30
21–25
16–20
11–15
6–10
1-5
0
Glucuronate_1
Malic acid
Glucose_2
Phenylalanine
Ornithine
Pyruvate+Oxalacetic acid
Palmitoleate
Orotic acid
Galactosamine_2
Proline
a-Sorbopyranose_1 (or Fructose_1)
Citric acid + Isocitric acid
Tagatose_1
Galacturonic acid_2
Valine(2TMS)
Glucarate
p-Hydroxybezoic acid
Galactosamine_1
Fumaric acid
Cystamine
Glutamine
Methionine
Lactic acid
Serine(3TMS)
Pantothenate
Asparagine
2-Aminoadipic acid
Lysine(3TMS)
Lyxose_1(or Xlylose_1)
trans-4-Hydroxy-L-proline
Urea
S-Benzul-L-Cysteine_1
N-Acetyl-L-Aspartic acid_1
Glutamic acid
Citrulline
Nonanoic acid(C9)
Fructose-6-Phosphate_4
Nicotinamide
Allose
Tryptophan
Inositol
Glucosamine_3
b-Glutamic acid
3-Hydroxy-Butyrate
Ketovaline_2
Acetoacetic acid
N-Methylethanolamine
Urea_1
Glucono-1,4-lactone
Glucosamine-6-Phosphate_3
Rhamnose_2
Hypoxanthine
5-Hydroxy-L-tryptophan_2
Aspartic acid
Lysine(4TMS)
Ketovaline(4TMS)
Dimethylbenzimidazole
Malonic acid
Pyroglutamic acid
Mannose_1
Glycerol
Glucuronate_2
Urocanate
meso-erythritol
Glycolic acid
5-Keto-D-Gluconate_1
Glyceric acid
Oxalate
HydroxyButyrate
Dopa
Benzoic acid
Lauric acid
1,5-Anhydro-D-glucitol
21–24 h
63–66 h
51–54 h
6–9 h
9–12 h
24 h LA
3 h LA

Figure S3. Metabolites used in prediction models that significantly predicted 3 h LA. Hierarchical clustering of metabolites based on their frequencies selected to build cross-validated prediction models for each time window of LA using R software (http://www.r-project.org/). LA, locomotor activity.

Term	*P*-value	FDR	Fold enrichment
Aspartate Metabolism	0.0115	1	
Malate-Aspartate Shuttle	0.0265	1	
Urea Cycle	0.0378	1	

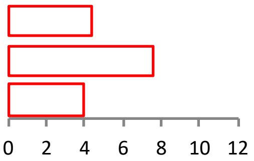

Figure S4. Pathway enrichment analysis for the metabolites used for constructing prediction model of 3 h LA at 63–66 h before sampling. The statistically enriched terms with raw *P*-value below 0.05 are shown.
